# Norwegian Soils and Waters Contain Mesophilic, Plastic-Degrading Bacteria

**DOI:** 10.3390/microorganisms9010094

**Published:** 2021-01-03

**Authors:** Colin Charnock

**Affiliations:** Department of Life Sciences and Health, Faculty of Health Sciences, Oslo Metropolitan University, Postbox 4, St. Olavs Plass, 0130 Oslo, Norway; colin@oslomet.no

**Keywords:** plastic-degrading bacteria, Norway, environmental samples, biochemical characterization, *Streptomyces* sp., *Rhodococcus* sp.

## Abstract

Plastic pollution has become one of the most critical environmental issues, as rapidly increasing production, compounded by persistence of plastic wastes in the environment, are outpacing efforts to keep ecosystems plastic-free. A switch to plastics more amenable to microbial attack is one of several possible responses. Against this background, the current study describes the isolation, enumeration and polyphasic characterization of plastic-degrading bacteria present in Norwegian terrestrial and aquatic habits. It shows that these bacteria are present in relatively high numbers, and that plastic-degrading capabilities are found in several taxa, most especially *Streptomyces*. Some isolates wereable to degrade several plastics. Notably, a *Rhodococcus* sp. and a *Streptomyces* sp. degraded, respectively, four and six of the eight plastics investigated and a number of other polymers relevant for plastic blends. The paper also has a methodological aspect, presenting various approaches for assaying plastic-degrading properties and a PCR/sequencing-based approach for the identification of potential polyethylene terephthalate-degrading genes. A candidate gene was detected in several *Streptomyces* isolates. The study shows that Norwegian environments are a rich source of bacteria with the ability to degrade bioplastics possibly representing a natural remediation capacity, as well as a potential source of useful enzymes.

## 1. Introduction

Plastics are a heterogenous collection of polymeric hydrocarbons. In common parlance they are synthetic molecules. However, plastic-like molecules occur naturally; bacteria produce the polyhydroxyalkanoate-type polymers polyhydroxybutyrate (PHB) and Poly(3-Hydroxybutyrate-*co*-3-Hydroxyvalerate) (PHBV) as storage molecules. Starch is commonly used in the production of bio-based biodegradable plastics, and cellulose- and starch-based materials can be used for some of the same functions as synthetic plastics, such as shopping and garbage collection bags. However, the main polymers that are currently considered of greatest importance to our economy are usually synthetic and derived from petroleum and similar sources. These include polyurethane (PUR), polyethylene (PE), polyamide (PA), polyethylene terephthalate (PET), polystyrene (PS), polyvinylchloride (PVC) and polypropylene (PP). Such plastics are highly stable and do not readily undergo degradation. About 80% of the global plastic-usage is of synthetic origin [1,2]. Generally speaking, there are many factors which contribute to the degradation rate of plastics, including molecular weight, melting temperature, glass transition temperature, the presence and types of additives and monomer chemistry [3]. Large-scale production and distribution, careless disposal methods and the inherent non-biodegradability of petroleum-based plastics are creating a major environmental concern, as plastics are accumulating in the environment and have become a threat to the biosphere. Every major aquatic environment on the planet is now contaminated with plastic wastes, and marine plastic litter in particular is considered a major challenge [4]. Microplastics and nanoplastics in aquatic environments are entering the food chain and ultimately end up in human intestines [4]. Several excellent reviews exist summarizing the extent and nature of contaminating plastics in natural ecosystems, and the possible if not probable negative effects they represent for the food chain and health [5,6,7]. 

High accumulation of low- or non-biodegradable plastics in the environment is driving an increased focus on readily biodegradable plastics, and the removal of plastics from the environment using microbes and microbial enzymes is a related avenue of recent research. Plastic production is a relatively new phenomenon, starting in essence in the 1950s–1960s; this is a very short time for the evolution of plastic-degrading enzymes, and the selective pressure for doing so in the presence of more bioassimilable substrates may be low. However, microbial enzymes capable of degrading or partly degrading a wide range of plastics are known [7]. Microbial biodegradation will often involve consortia of multiple species, some of which are capable of degrading high molecular weight polymers into simpler forms (multipers, dimers) using exoenzymes; these are then broken down by other microbes in the community [7,8]. Carboxylesterases (E.C. 3.1.1.1), lipases (E.C. 3.1.1.3) and cutinases (E.C. 3.1.1.74) are those classes of enzymes chiefly associated with the hydrolysis of plastics [9]. Most biodegradable plastics are polyesters (e.g., the polyhydroxyalkanoates, polycaprolactone (PCL), poly(ethylene succinate) (PES), poly(1,4-butylene succinate) (PBS), Poly(L-lactide) (PLLA) and polybutylene succinate-*co*-adipate). Polyesters can, in one scheme, be further categorized into three groups: those bracketed above are examples of aliphatic polyesters and are hydrolyzed by various ester-bond hydrolases. In addition, there are aromatic polyesters, e.g., polyethylene terephthalate (PET), which are the least biodegradable [10,11]. In an attempt to combine good material properties with biodegradability, aliphatic–aromatic copolyesters, such poly (butylene adipate-*co*-terephthalate) have been developed [10,11]. Aliphatic polyesters alone or as copolymers (to improve processing and end-point properties) have been used for many applications such as shopping bags, film production in agriculture and as coatings. Some have been approved for in vivo applications, including controlled drug delivery systems in bone tissue engineering [12]. Polyesterase acting on aromatic polyesters (primarily PET) was first reported for *Thermobifida fusca* [13]. Although several PET-degrading enzymes are now known [14], there is currently only a single report of the utilization of this plastic for bacterial growth. In 2016, the first PET-degrading microbe also capable of utilizing this synthetic plastic as a source of carbon and energy was isolated from a PET-contaminated soil [15]. The bacterial strain *Ideonella sakaiensis* 201-F6 was shown to grow on low-crystallinity PET films. Two α/β-hydrolase fold enzymes, PETase (a unique cutinase-like enzyme) and MHETase, work together to degrade PET in two steps. The residues constituting the substrate binding site in enzymes with known PET-hydrolyzing ability are almost conserved [9,14,16,17]. However, PETase has a number of unique features near the catalytic center, and the active site of PETase is wider than that of the other PET-hydrolyzing cutinases. Presumably because of these features, PETase is 5.5–120-fold more efficient than previously reported PET-hydrolyzing homologs [9]. 

The present study, which is the first of its kind in Norway, has as its main aim to isolate, identify and characterize the plastic-degrading potential of bacteria from environmental samples in Norway. This will provide an insight into the types and distribution of these bacteria, and to some extent their potential to process plastics entering the environment. It may also identify novel enzyme activities with useful plastic-degrading capabilities. 

## 2. Materials and Methods 

### 2.1. Samples and Isolation and Enumeration of Bacteria

The majority of strains originate from a systematic investigation of plastic-degrading bacteria in nine soil, sediment and water samples (Table 1). The approach used here was as follows: samples (about 10 g) were taken with a sterile spatula and transferred to a 50 mL sample tube with a screw cork. Samples were transported cold and analyzed within 24 h. All samples were taken in the period January to March, 2019. Salient information on the nine samples is given in Table 1. 

The following approach was developed to isolate and detect plastic degrading bacteria: samples 1.0 ± 0.1 g wet weight and a spatula of 2 mm glass beads were transferred to a 15 mL centrifuge tube (Corning™ from Sigma-Aldrich, St. Louis, MO, USA, Catalogue number CLS430055). Thereafter, Maximum Recovery Dilutant (MRD; Oxoid, Hampshire, UK, Catalogue number CM0733) was added to give a total volume of 10 mL. The mixture was then vortexed vigorously (Test Tube Shaker, Labworld-online.com) using three 1 min bursts and intermittent cooling. Subsequently, tubes were placed in a vertical position for 10 min to allow the largest particles to sink. One mL of the top layer was transferred to a new tube and serially diluted 1/10 a further six times with MRD. From each dilution, a sample (0.1 mL) was transferred to R2A (Oxoid, Hampshire, UK. Catalogue number CM0906) and incubated at 22 ± 2 °C for 14 days. After incubation, the dilution (plate) for each sample showing 50–100 colonies or the number nearest to this, was chosen for interpretation and screening for plastic-degrading bacteria. The number of different colony types judged visually based on color, shape, pattern and consistency were noted. In the initial screening, a portion of each colony was spotted onto agars containing the following plastics: PHBV, PHB, PCL, PES, PBS and PLLA (see details in 2.2). Plates were incubated at 22 ± 2 °C and examined for appearance of zones of clearing around colonies over a six-week period. The study also includes two strains isolated previously from a soil in the vicinity of a small industrial unit (59°24′49.5″N 10°41′32.3″E) producing plastic products. Here, soil particles were sprinkled onto PHB-containing agar, and two strains with PHB-degrading capabilities were subsequently isolated from zones of clearing.

### 2.2. Plastic-Containing Agar

The test media consisted of two layers (each made of about 20 mL agar solution in 9 cm petri dishes): a bottom layer consisting of vitamins and salts overlayed with an agar containing the test plastic. The bottom layer contained 0.01 g casamino acids (Bacto™, BD Diagnostics-TriPath, Burlington, USA, Catalogue number 223120), 0.01 g yeast extract (Sigma-Aldrich, St. Louis, MI, USA, Y1625), 2.27 g M9 minimal salts (Sigma-Aldrich, m-6030) and 15 g washed agar-agar (Merck, Kenilworth, NJ, USA, Catalogue number 101614) in 1000 mL ion-exchange purified water. After sterilization by autoclaving and cooling to circa 60 °C, 1 mL Trace Metal Mix (Sigma-Aldrich, St. Louis, MI, United States, Catalogue number 92949), 2.4 mg Mg^2+^ (from 10 mg/mL filter sterilized stock) and 0.4 mg Ca^2+^ (from 10 mg/mL filter sterilized stock) were added and plates were poured. The top agar layer contained 0.1–0.2% (*w*/*v*) plastic in 15 g/L twice washed agar-agar (Merck, Darmstadt, Germany, Catalogue number 101614) in 1 × PBS. Two different approaches, dependent on the plastic form (powder or pellets), were used to include the plastic in the top agar: for fine powders, a suspension was made in 10 × PBS at pH 7.2, the suspension was then added dropwise to agar (in water) melted at 60 °C with vigorous stirring to achieve 0.1–0.2% *w*/*v* plastic and 1.5% agar in 1 × PBS. To achieve a homogenous suspension in 10 × PBS, the plastic powder was added slowly to the liquid placed in an ultrasound-generating water bath (S30 Elmasonic, Elmasoni). Repeated pipetting with a glass pasteur pipette was used to break up small clumps of powder.

Plastic pellets were dissolved in dichloromethane (5 mL) and added dropwise under sonication, at 40% amplitude with 45 s on/15 s off pulses (Vibra-Cell VCX130, Sonics, Seattle, WA, USA), to 200 mL molten, autoclaved agar in 1 × PBS and containing 0.1 g/L N-Lauroylsarcosine (Sigma-Aldrich, St. Louis, MO, USA, Catalogue number L5777). 

The following eight plastics were included in the study: poly[(R)-3-hydroksybutyrate], PHB (fine powder; Sigma-Aldrich, 363502); poly(3-hydroksybutyrate-co-3-hydroksyvalerate), PHBV (fine powder; Materials Gateway, UK, sold as ENMAT Y1000P); poly(ethylene succinate), PES (Pellets, average molecular weight = 10,000; Sigma-Aldrich, St. Louis, MO, USA, Catalogue number 182036); poly(1,4-butylene succinate), extended with 1,6-diisocyanatohexane, PBS (pellets; Sigma-Aldrich, Catalogue number 448028); polycaprolactone, PCL (Powder/flakes, MW 50,000; Polysciences 25090, Warrington, PA, USA); poly(L-lactide), PLLA (Fine powder, MW ~1600–2400; Polysciences 18580-1, Tyskland); Resomer^®^ RG 502, Poly(D,L-Lactide-*co*-Glycolide), RES (Fine, amorphous powder: lactide:glycolide 50:50 and ester terminated, MW 7000–17,000); polyethylene terephthalate, PET (amorphous sheet; Goodfellow, ES303010, London, UK).

### 2.3. PCR Amplification and Sequencing Studies

Identification based on the 16S rDNA gene: The PCR reaction mixture (50 μL) contained 3 μL of 25 mM MgCl_2_ (Promega), 1 μL dNTPS 10 mM (Promega), 10 μL HotStart DNA polymerase buffer, 0.2 μL HotStart DNA polymerase 5 U/μL (Promega), 0.25 μL of each of primers 27f (AGA GTT TGA TCA TGG CTC A) and 1492r (TAC GGT TAC CTT GTT ACG ACT T) {100 μM stock of standard à la carte sequencing primers from MWG Eurofins} and PCR-grade water to 50 μL. To provide the template, a flame-sterilized steel pin was touched onto a bacterial colony and the pinpoint of material was transferred to the reaction mix.

PCR conditions were: one cycle at 95 °C (10 min) followed by 32 cycles of 95 °C/min, 52 °C/45 s and 72 °C/1.5 min. This was followed by a final elongation of 72 °C/12 min. Products were checked for purity by agarose electrophoresis and DNA concentrations were measured using Qubit™ dsDNA BR Assay Kit (Thermo Fisher Scientific, Waltham, MA, USA). PCR products were sequenced on both strands at least twice. Sequencing was performed by a commercial laboratory (MWG Eurofins) using the PCR primers. Sequences were aligned using Clustal Omega [18], and the consensus region of overlap was used for purposes of identification. Sequences were analyzed and assigned to taxa using the RDP Naive Bayesian rRNA Classifier Version 2.11 (RDP rRNA training set 18) with the default 80% confidence threshold [19]. In addition, BLAST [20] was used to search the NCBI database. The curated ‘Reference RNA sequence’ setting in BLAST was used to assign the sequences to a named taxon.

Touchdown PCR amplification and sequencing of genes potentially encoding PET-hydrolyzing enzymes: The reaction mixture (50 μL) contained 3 μL of 25 mM MgCl_2_ (Promega, Madison, WI, USA), 1 μL dNTPS 10 mM (Promega, Madison, WI, USA), 10 μL HotStart DNA polymerase buffer, 0.05 μL HotStart DNA polymerase 5 U/μL (Promega, Madison, WI, USA), 0.2 μL of the forward primer (GTCATCACCATCGACACCA) and 0.2 μL of the reverse primer (GTAGCG(G/C)GTGTCGTTGTC). A touchdown PCR was performed with the following specifications: An initial denaturation 95 °C/10 min was followed by 36 cycles in which the annealing temperature was lowered from 65 °C by 1 °C in each round until it reached 55 °C (36 cycles: 95 °C/30 s; 64–55 °C/30 s; 72 °C/60 s). A final elongation step of 72 °C/5 min completed the reaction. Quality control and measurements of DNA concentration were carried out as described above. The primers were designed based on alignments of the PET-hydrolyzing genes of Gram-positive (*Thermobifida fusca*) and Gram-negative bacteria (*I. sakaiensis* NBRC 110686, *Acidovorax delafieldii*) presented with references in Joo et al. [14], and amplify a region of approximately 480 bp in these genes. The PCR product was checked for an amplicon of expected size by gel electrophoresis. PCR products were sequenced on both strands at least twice. Sequencing was performed by a commercial laboratory (MWG Eurofins, Ebersberg, Germany) using the PCR primers. *I. sakaiensis* NBRC 110686 was used as the control for PCR, and its amplicon was also sequenced to check the fidelity of the reaction. Sequences were aligned using Clustal Omega [18] and the consensus region of overlap was used for further analysis. Open reading frames were generated based on the sequences by comparison with the PET-hydrolase enzyme from *I. sakaiensis*. Similarity searches on the sequences were performed using BLASTP [20] to search the non-redundant protein sequence library at NCBI [21]. Additional searches were performed using FASTA [22] to search the manually annotated section of UniProtKB [23].

The ORF with significant similarity to the control PETase sequence from *I. sakaiensis* was aligned with putative PET-hydrolysis genes given in [14]. Sequences were aligned using Clustal, and key residues were identified across the sequences by reference to the literature (see Results and Discussion). A phylogenetic tree for PETase and PETase-like enzymes was generated using the Phylogeny.fr [24,25] online tool (www.phylogeny.fr/) in the a la carte mode: reference sequences were trimmed manually to approximately equal size to those of the isolate sequences, and aligned using MUSCLE (v3.8.31) in the program package [26]. After alignment ambiguous regions (i.e., containing gaps and/or poorly aligned) were removed with Gblocks v0.91b [27]. The tree was constructed using the maximum likelihood method [28], implemented with the approximate likelihood-ratio test (aLRT) setting for branch support [29] included in the pipeline PhyML (v 3.0) program. The default WAG substitution model for amino acids was chosen to account for rate heterogeneity across sites [30]. Internal branch reliability was assessed using the bootstrapping method (100 bootstrap replicates). Graphical representation of the phylogenetic tree was achieved using the workflow TreeDyn tool [31]. 

### 2.4. Broth-Based Investigation of PET-Degrading Activity 

In order to provide an additional indication of possible PET-hydrolysis, small pieces (approximately 3 × 3 mm) of amorphous plastic hung from fishing line were suspended in a dilute growth medium (g/L: 0.2 casamino acids; 1.26, yeast nitrogen base (Merck, Kenilworth, NJ, USA, Y1251); 0.04 yeast extract) seeded with a pure culture of *I. sakaiensis* NBRC 110686 or sample isolates. Bottles were observed over a period of 6 weeks (room temperature, without shaking) with occasional changes of medium. Development of growth and discoloration on the plastic were looked for. The technique is basically the same as what was used in the original isolation of *I. sakaiensis* [15].

### 2.5. API^®^ZYM (bioMérieux Inc.)

The *Rhodococcus* sp. was grown on BUG agar plates (Biolog Inc., Hayworth, CA, USA) at 30 ± 1 °C for 24 h. A thick suspension of isolated colonies was made in 0.85% NaCl and this was used to create the inoculum (McFarland 5-6) exactly as described in the product insert. Sixty-five µL of the inoculum was pipetted into each cupule and plates were incubated at 31 ± 1 °C. In each round of testing (two in total), plates were interpreted after 20 h incubation.

The *Streptomyces* sp. was grown on oatmeal agar (Sigma-Aldrich, St. Louis, MO, USA, Catalogue number O3506) for 7 days at 31 ± 1 °C. A thick hyphal/spore suspension was made in 0.85% NaCl and this was used to create the inoculum. Thereafter, the same procedure as that used for *Rhodococcus* was followed. 

### 2.6. API^®^50CH (bioMérieux Inc.)

*Rhodococcus/Streptomyces* were grown as for the API^®^ZYM test. A suspension in API 50 CHB/E medium (the inoculum) was made exactly as described in the product insert. Plates were inoculated without oil overlay in the cupule at 31 ± 1 °C and read at 48 h and 6 days. The system was quality-controlled using *Paenibacillus polymyxa*, as suggested in the package insert.

### 2.7. Assays for Additional Enzyme Activities

Sodium carboxymethyl cellulose (CMC) agar for detection of cellulase activity: 50% R2A agar (Oxoid) was amended with CMC (Sigma-Aldrich, St. Louis, MO, USA, Catalogue number 419338) at 0.5% *w*/*v*. Plates were inoculated to achieve well-isolated colonies. After incubation at 31 ± 1 °C for 72 h, plates were flooded with 0.1% Congo Red (Sigma-Aldrich, St. Louis, MO, USA, Catalogue number C6767) for 15–20 min. Plates were then rinsed carefully with 1 M NaCl. The NaCl was decanted and replaced, and plates were then agitated gently for 20–30 min. The NaCl was again removed. Zones of clearing round individual colonies against a red background of none-hydrolyzed CMC was taken as an indication of cellulase activity. *Paenibacillus polymyxa* DSM 365 and 372 were used as positive controls.

Tributyrin agar for the detection of esterase/lipase activity: 50% R2A agar (Oxoid, Hampshire, UK) was amended with tributyrin (Sigma-Aldrich, St. Louis, MO, USA, W222305) at 1% *v*/*v* with mixing. Plates were inoculated to achieve well-isolated colonies. After incubation at 31 ± 1 °C for 72–96 h, plates were examined for zones of clearing around colonies indicating esterase/lipase activity. *Burkholderia cepacia* DSM9421 and *Pseudomonas aeruginosa* DSM 1128 were used as positive control organisms.

Olive oil—Rhodamine B agar for the detection of lipase activity: R2A agar (Oxoid, Hampshire, UK) was amended with olive oil (Sigma-Aldrich, O1514) as follows: 30 mL of olive oil was emulsified (Vibra-Cell VCX130, Sonics) into 50 mL water containing 250 µL of Tween 20 (Sigma-Aldrich, St. Louis, MO, USA, P1379). The emulsion was autoclaved, cooled to 60 °C and amended with 20 mL of a filter sterilized Rhodamine B solution (1 mg/mL). Then, 50 mL of this mixture was added with stirring into 450 mL R2A agar melted at 50 °C. Plates were prepared and inoculated to achieve well-isolated colonies. After incubation at 31 ± 1 °C for 72–96 h, plates were examined for orange fluorescent zones emanating from colonies and also zones of clearing around colonies, indicating lipase activity. *Burkholderia cepacia* DSM9421 and *Pseudomonas aeruginosa* DSM 1128 were used as positive control organisms. 

Coconut oil—Rhodamine B agar for the detection of lipase activity: Coconut oil-agar was made essentially as described for olive oil agar, except that 20 mL of melted coconut oil (Green choice, Vantaa, Finland) was used. Analysis and interpretation were as described for olive oil agar.

Starch agar for the detection of α-amylase activity: 50% R2A agar (Oxoid, Hampshire, UK) was amended with starch (Sigma-Aldrich, 03967) at 0.5% *w*/*v*. Plates were inoculated to achieve well-isolated colonies. After incubation at 31 ± 1 °C for 72 h, plates were flooded with Gram’s iodine. Colorless zones surrounding the colonies against a blue/purple background of non-hydrolyzed starch was taken to indicate amylase activity. *Paenibacillus polymyxa* DSM 365 and 372 were positive controls.

Skim milk powder agar for the detection of protease activity: 50% R2A agar (Oxoid, Hampshire, UK) was amended with skim milk powder (Sigma-Aldrich, 70166) at 2.8% *w*/*v*. Plates were inoculated to achieve well-isolated colonies. After incubation at 31 ± 1 °C for 72 h, plates were examined for zones of clearing around colonies indicating protease activity. *Burkholderia cepacia* DSM 9241 and *Pseudomonas aeruginosa* DSM 1128 were used as positive control organisms

DNase agar: Commercially available plates containing 0.2% w/v deoxyribonucleic acid (Oxoid, Hampshire, UK, Catalogue number CM0321) were inoculated to achieve well-isolated colonies. After incubation at 31 ± 1 °C for 72 h, plates were flooded with 1N HCl and allowed to stand for 5 min. Zones of clearing in the agar around colonies was taken to indicate DNase activity. *Staphylococcus aureus* strains ATCC 25923 and DSM 799 were used as positive control organisms.

Hemolysis on sheep blood agar: Commercially available Columbia agar plates containing 7% sheep blood (Oxoid, Hampshire, UK, Catalogue number PB5039A) were inoculated to achieve well-isolated colonies. After incubation at 31 ± 1 °C for 72 h, plates were examined for hemolysis around single colonies. *Streptococcus pyogenes* ATCC 19615 (producing beta hemolysis) was used as a positive control. 

Test for beta-lactamase activity: Production and activity of beta-lactamase activity was tested for using commercially available discs containing the chromogenic cephalosporin Nitrocefin (Remel, San Diego, CA, USA). Using the kit protocol, a color change to pink/red was taken as an indication of enzyme activity. *Escherichia coli* ATCC 35218 and *S. aureus* DSM 2569 were used as positive controls.

Accession numbers: 16S rDNA sequences: MW269812-22. Putative PET-hydrolases: MW281313-17.

## 3. Results and Discussion

### 3.1. Numbers, Identities and Range of Activities of Plastic Degrading Bacteria

**Figure 1:**
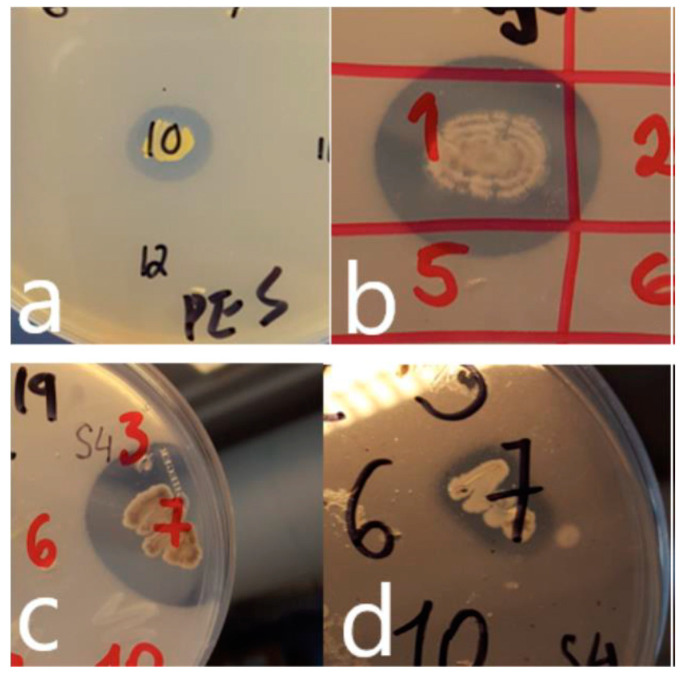
Growth of isolates and production of zones of clearing on plastic-containing agars (**a**) *Rhodococcus* sp. (isolate vii; Table 2) on PES-agar (**b**) *Streptomyces* sp. (isolate iv; Table 2) on PHB-agar (**c**) *Streptomyces* sp. (isolate viii; Table 2) on PHB-agar (d) *Streptomyces* sp. (isolate viii; Table 2) on PCL-agar.

For the nine environmental samples forming the core of the study, the CFU/g wet weight ranged from 5 × 10^4^ (sample 3) to 6 × 10^7^ (sample 1). A single dilution/agar plate (showing between 50–100 colonies) was chosen for further investigation. The number of different colony-classes on this plate was visually adjudged to range from 6 (sample 3) to 19 (sample 7). In total, 88 different colony types across all nine samples were recognized, and a representative was transferred to a range of six plastic-containing agars. Figure 1 shows some typical results from the agar screening tests. Six of the nine samples contained one or more plastic-degrading bacteria. Colonies originating from samples 2, 6 and 8 did not produce clearing on any agars. In total, 9 of the 88 colony types showed clearing on one or more of the plastic-containing agars. Summary data on their degradative properties are shown in Table 2 (isolates designated iii-xi). In addition, two isolates (i-ii; Table 2) from a previous, preliminary study were also characterized. Subsequently, activity against additional plastics and other polymers were tested for. Attempts were not made to determine the absolute numbers or proportion of different plastic degrading bacteria arising from each sample. However, as mentioned, of the 88 different colony types tested, 9 (about 10%) showed the ability to degrade one or more of the 8 plastics used in the initial screening. 

Table 3 gives a detailed presentation of the sequencing-based identifications of the 11 isolates based on similarity searches of partial 16S rDNA gene sequences.

In agreement with previous investigations [1,32,33,34], the present study shows that the ability to degrade one or more plastic is a fairly common trait and is found across a range of taxa of easily cultured species present in environmental samples. None of the 11 plastic-degrading isolates had effect on PLLA, whereas 9 caused clearing in PHBV, and this plastic was the one that was the most commonly degraded by the bacteria (Table 2). Based on similar clear-zone techniques, others have looked at populations of bacteria in different environments. Urbanek et al. [1] reported the following order of degradation of plastics by artic microorganisms: PCL > PBS > PLA (isomeric form not specified). The same order was seen for microbes from agricultural soil in Thailand [33]. Looking at microorganisms in different ecosystems, Nishida et al. [34] found high and approximately equal numbers of PHB- and PCL-degraders. These results are all similar to those of the present work. The complete order of biodegradation in the present study, when based on the number of different isolates producing clearing in plastic-containing agar, was as follows PHBV > PHB > PCL > PBS =PES >PLLA (Table 2). The general agreement with previous studies, which together cover a wide range of clean environments, might suggest this order reflects a general tendency in environmental bacterial isolates. If so, this could help inform future choices on green plastics with respect to consequences on entering the environment.

There were no bacteria that degraded PHB and not PHBV, while the reverse was not the case (Table 2). The naturally occurring aliphatic polyesters PHB and PHBV are accumulated as carbon and energy storage materials in various microorganisms. PHB is the most common and simplest form of PHA found in bacteria. PHBV is the copolymer of 3-hydroxybutyrate (3-HB) and 3-hydroxyvalerate (3-HV) and is synthesized in bacteria especially when the growth medium contains organic acids [35]. Higher biodegradability of PHBV than PHB has been reported previously [36]. However, Boyandin et al. [37] found for isolates from Vietnamese soils that the opposite was the case. It should be noted that comparisons of this sort must be seen in the light of several other extenuating factors, such as molecular weight, degree of crystallinity and, in this instance, the percentage of the copolymer. Degradation in the cell would be required for mobilization as substrates for growth, and this could explain why PHA-degradation was that most commonly found in the present and other studies. Owing to their biodegradability, and physical properties similar to most of the synthetic plastics, PHAs are considered as a green substitute for petroleum-derived plastics. However, the limiting physical properties of PHB, such as brittleness, high crystallinity and instability during the melting stage, hinder its wide-spread application [38]. PHBV has superior properties such as better thermal behavior, plasticity, toughness and biodegradability, making it more attractive in the bioplastic market [39]. As discussed above and as seen in Table 2, both PHB and PHBV are readily degraded by a broad spectrum of taxa. PCL is a biodegradable, synthetic semi-crystalline aliphatic polyester made by the ring-opening polymerization of ε-caprolactone. It is a fossil-based biodegradable polyester which is reportedly degraded by both aerobic and anaerobic microorganisms [3]. It is used for blood bags, catheters and packaging materials [40]. The present and previous reports show in addition that PCL is readily hydrolyzed by environmental strains, adding to its potential suitability as a green alternative. PBS and PES are other types of aliphatic polyesters that are known to be degraded by microorganisms. However, published reports on the degradation of these polymers are sparse. The distribution of PES degrading microorganisms in the environment has been suggested to be low compared to PHB- and PCL-degraders [3], and the same tendency was seen in the present study. The ability to degrade the aliphatic polyesters PES and PBS was found only in the single *Rhodococcus* isolate and in one of the *Streptomyces* isolates (isolate viii; Table 2). These isolates were also two of only four that degraded PCL (Table 2), and only these and one other isolate were able to degrade Resomer^®^ RG 502, Poly (D,L-Lactide-*co*-Glycolide (Table 2). Resomer^®^ RG 502 is one of series of biodegradable polymers for medical device applications research. These copolymers have been researched for a wider range of applications than any other type of biodegradable polymers. They are polyesters that biodegrade in the body by simple hydrolysis of the ester backbone to non-harmful and non-toxic compounds and can be used in for example drug delivery systems (see Introduction). Thus, although none of the isolates had an effect on PLLA, several could degrade its useful copolymer. Ecological studies on the abundance of PLLA-degrading microorganisms in different environments have confirmed that PLLA-degraders are not widely distributed [3,41,42,43]. 

Aliphatic polyesters, e.g., cutin and suberin, also exist in nature as water-insoluble polymeric materials occurring on higher plants. It has been reported that some named fungal phytopathogens formed clear zones on emulsified PCL agar plates. This could involve the serine hydrolase, cutinase. It is suggested that PCL is an analogue of natural products of polyesters such as cutin, and as such the possibility of its use as a ‘model’ substrate for PET hydrolase and cutinase activities has been raised [44]. This is based on the suggestion by Yoshida et al. [15] that PETase has close sequence identity to bacterial cutinases, with *Thermobifida fusca* cutinase being the closest known structural representative. 

The closest named relative of the PCL-hydrolyzing *Rhodococcus* isolate (Table 3) is *Rhodococcus fascians*. Colony pigmentation (a dull yellow) is a further similarity. *R. fascians* is a well-known phytopathogen which infects a wide range of plants, initiating the formation of leafy galls [45]. If the isolate is a phytopathogen, its wide array of polymer-degrading enzymes may be an aid to colonization of plants. Phytopathogens should be a focus of future screening studies aimed at finding new plastic-degrading species and enzyme specificities. Furthermore, the study conducted by Urbanek et al. [1] in comparable cold climates to that of Norway, documents 113 bacterial and 8 fungal species which could degrade variously poly (butylene succinate-co-adipate) (PBSA), PBS, PCL and PLLA. Of these, *Pseudomonas* spp. and pertinently *Rhodococcus* spp. were found to have the highest degradation capacity. 

The closest named relative to the multi-polymer degrading *Streptomyces* sp. (Table 3) is *S. brevispora*, which forms a distinct clade based on sequencing of the 16S rDNA gene with *S. laculatispora* and *S. druzdowski* [46]. However, the sequencing-based delineation from multiple *Streptomyces* species is not particularly significant in terms of percentage-identity. *Streptomyces* spp. are well-known for the production of a great range of enzymes and secondary metabolites, most particularly antibiotics. Perhaps unsurprisingly, there are several publications relating to plastic-degrading properties in the genus [47,48]. With respect to degradation of plastics and other non-plastic polymers, the *Streptomyces* isolate had the most wide-ranging biodegradation profile (Table 2). This theme is returned to below in terms of a possible PET-degradation potential. 

### 3.2. Additional Characterizations of Multi-Polymer Degrading Rhodococcus and Streptomyces 

Based on their plastic-degrading profiles, it was most interesting to single out for further study the *Rhodococcus* isolate and *Streptomyces* isolate viii (Table 2). A series of additional biochemical tests to establish metabolic profiles (particularly hydrolytic enzymes) were performed.

#### 3.2.1. API^®^ ZYM and Correlations to Observed Lipid/Polysaccharide Hydrolysis

As discussed in the Introduction section, esterases and lipases are among the major classes of enzymes involved in plastic degradation. The API-ZYM kit assays for 20 enzyme activities, with several tests dedicated to distinguishing substrate C-chain lengths for esterases and lipases. The use of tributyrin, a short-chain fatty acid triglyceride in agar plates, will detect activities of esterases and true lipases. The use of triglycerides with long-chain fatty acids (FA) such as olive oil instead is more selective for lipases, because activity towards substrates with fatty acid chains > C10 is a characteristic of these enzymes [49]. Cutinases have been categorized as between esterases and true lipases, because they are reported to have higher affinities for short-chain to middle-chain fatty acid ester substrates with chain lengths up to C8 or C12 [50]. Because of this, established lipase-specific screenings with olive oil agar may miss lipolytic enzymes with additional polyesterase activity [49]. The application of coconut oil that contains, in contrast, a large portion of C6-C14 FA esters [51] may bridge the gap between tributyrin and olive oil. Both *Streptomyces* isolate viii and *Rhodococcus* were able to hydrolyze the 4-carbon 2-naphthyl butyrate in the API ZYM test, which is the expected result for esterases and true lipases. This finding was is in line with the observation that both isolates also gave zones of clearing on tributyrin agar (Table 2). The *Rhodococcus* sp. gave strong hydrolysis of the kit C8-substrate, but a much weaker result was seen with *Streptomyces.* Coconut oil contains a large proportion of C6-C14 fatty acid esters and *Rhodococcus* also produced zones of clearing in this agar. As mentioned, the major fatty acids in olive oil are longer than in coconut oil (typically C16–C18) and neither *Rhodococcus* nor *Streptomyces* produced visible zones of hydrolysis on olive oil agar. However, a weak lipase activity on the API^®^ ZYM C14-substrate was obtained for *Rhodococcus*, confirming the presence of enzymes with longer-chain specificities. Thus, both isolates, particularly *Rhodococcus,* showed the ability to produce classes of enzymes (esterases, lipases) also associated with plastic-degradation. Furthermore, the results also show that the agar hydrolysis test and kit enzyme tests are mutually informative.

*Streptomyces*, but not *Rhodococcus*, produced hydrolysis on CMC and starch agars (Table 2), indicating cellulase (endo-beta-1,4-glucanase) and α-amylase (1,4-alpha-D-glucan glucanohydrolase) activity, respectively. In accord with this, the API ZYM kit detected some activity of both beta and alpha glycosyl hydrolases. This is a relevant finding with respect to a potential for biodegradation and bioremediation of commonly used starch/cellulose-based plastic-products and blends (see Introduction). 

In addition to the API ZYM results for esterase/lipase and amylases discussed above, other salient findings with respect to the present study and polymer hydrolysis, were that neither isolate hydrolyzed the kit substrates designed to indicate the activity of trypsin and α-chymotrypsin. This is in accord with the lack of hydrolysis on skimmed milk agar for the *Rhodococcus* isolate. However, the *Streptomyces* isolate produced clearing in the agar. This seeming discrepancy could be explained by the presence of other bacterial proteases.

#### 3.2.2. API^®^50CH

Supporting information on the general metabolic potentials of the isolates was obtained using the API^®^50CH assay. The results from the analysis suggest that even after prolonged incubation, relatively few carbohydrates were metabolized by *Rhodococcus* under the conditions of the test, perhaps suggesting a metabolism more tailored to non-carbohydrate substrates. Conversely, a wider range of substrates (about double that for *Rhodococcus*) were definitively or probably metabolized by *Streptomyces*. With respect to *Rhodococcus*, glycerol, L-arabinose, D-glucose, D-fructose, D-mannose, D-mannitol, D-sorbitol, D-arabitol and possibly D-saccharose were metabolized. This pattern is highly similar to that recorded for *Rhodococcus luteus* [52], which is a close phylogenetic neighbor to the isolate in this study [53]. 

#### 3.2.3. Additional Enzyme Tests

*Hemolysins: Streptomyces* produced zones of partial clearing around individual colonies. Colonies of *Rhodococcus* did not produce any visible effect in the surrounding agar. The best described *Streptomyces* hemolysin is the S-hemolysin produced by *Streptomyces coelicolor* [54]. It has been suggested that that this may assist the cell in nutrient and specifically iron uptake. The hemolysin produced by the *Streptomyces* isolate in the present study could potentially have a similar function and is a further example of the wide array of extracellular factors produced by this bacterium.

Test for beta-lactamase activity: *Streptomyces* produced faint but definite pink coloration (score +: against +++ for control strains) on discs indicating enzyme activity. No change in color was observed with *Rhodococcus*, indicating no enzyme activity.

### 3.3. Presence of Putative PET-Hydrolase Genes 

**Figure 2:**
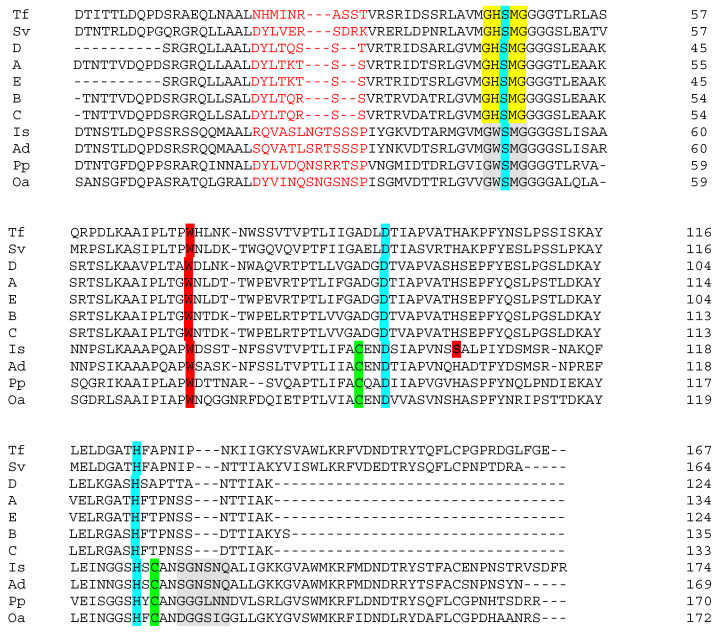
Alignment of putative PET-degrading enzymes. Is, Ad, Pp, Oa, Tf and Sv are representations of the enzymes from *Ideonella sakaiensis* (PETase), *Acidovorax delafieldii, Pseudomonas pseudoalcaligenes, Oleispira antarctica, Thermobifida fusca* and *Saccharomonospora viridis*, respectively (see [14] and references therein). A, B and C are enzymes from *Streptomyces* isolates iv, viii and xi (Table 2 in the present work). Sequences C and D are from *Streptomyces* spp. isolated in a separate study of PCL-degrading bacteria. Color code: Residues marked in light red form a region close to the catalytic site, which is shorter in the *Streptomyces* enzymes than in PETase. The residues marked yellow/grey form the motif G-X1-S-X2-G, which is highly conserved in serine hydrolases. Residues S,D,H highlighted in turquoise are the canonical catalytic triad of cutinases. Cysteine (C) residues, representing a potential for disulfide bond formation, are marked in pale green. The extended loop is highlighted in grey. W marked in dark red is present in all sequences and is probably involved in binding of substrate. However, the serine, S, also marked in dark red, is found exclusively in PETase. Serine at this position allows rotation of W (‘wobble’), which has been shown to be important for effective substrate binding by the *I. sakaiensis* PETase

All of the isolates from the present study were analyzed using a touchdown PCR/sequencing- based approach for the presence of putative PET-degrading genes. It was hypothesized that if a single, similar-sized product to the control strain was obtained with these primers, it might be indicative of a PET-degrading potential. Only the *I. sakaiensis* control and three of the isolates (iv, viii, xi; Table 2), all of which were *Streptomyces*, produced a product. In each instance, a single product of the expected size was obtained. A large number of laboratory strains representing many genera (results not shown) did not give rise to PCR-products suggesting fidelity in the analysis. Given the relative specificity of the system and the correct predicted size of the product, ORF of the sequences were generated. The ORF with significant similarity to the control PETase sequence was identified. It should be noted that isolates ii, iii and v, which are also *Streptomyces*, did not produce a product, indicating that the targeted gene is probably not present in all members of the genus. In a parallel study (unpublished results) looking at the presence of PCL-degrading bacteria in the environment, five isolates resembling *Streptomyces* were found. Of these, two (both of which were reliably assigned at the genus level by 16S rDNA sequencing) also produced a correctly sized PCR-product. The PCR-product was sequenced for both isolates and the PETase-like ORF was generated and is included. Figure 2 shows alignments of PETase and other PET-hydrolyzing enzymes and is adapted from Joo et al. [14] to include the *Streptomyces* ORF from this report. Key residues related to catalysis are highlighted in the figure [9,14,16,17].

**Figure 3:**
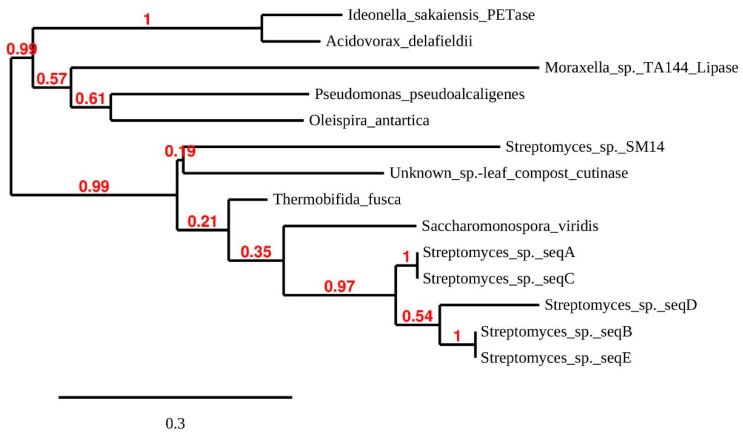
Maximum likelihood phylogenetic tree of putative PET-hydrolyzing enzymes and some structural homologs. The tree is based on sequences aligned in Figure 2 and additional ones: *Streptomyces* sp. SM14 (from a marine sponge) alpha/beta hydrolase (DAC80635). Unknown species: leaf compost cutinase (G9BY57). *Moraxella* sp. lipase (P19833) for rooting of the tree. Tree branch support values are shown in red at the nodes. The scale bar indicates the average number of amino acid substitutions per site.

Figure 2 shows that *I. sakaiensis* PETase, other potentially PET-hydrolyzing enzymes (previously reported) and *Streptomyces* enzymes A–E share high general sequence similarity, and a number of key elements related to catalysis. A relatively high number of residues that comprise the substrate-binding pocket are conserved or semi-conserved in all the sequences shown in the figure. It is beyond the scope of this discussion to look at these in detail. However, the reader is referred to Figure 2 in Chen et al. [9] for just such a comparison of general primary structure similarity. The active site of α/β hydrolase superfamily proteins, such as cutinases, esterases and lipases as well as *I. sakaiensis* PETase, contains the highly conserved serine hydrolase sequence -(G)-X1-(S)-X2-(G). This is present in all of the sequences (Figure 2), and in the alignments, two subclasses (marked in yellow and grey) are recognized based on the identities of X1 and X2. Cutinases employ a canonical catalytic triad to carry out ester bond hydrolysis consisting of Ser(S)-His(H)-Asp(D), and this is also seen in all of the sequences (turquoise shading) [17]. However, the *I. sakaiensis* PETase is known to form an additional disulfide bond between the Cys(C) residues shown in green in the vicinity of the active site. This links two loops that harbor the catalytic acid (D) and base (H) of the triad. This possibility is lacking in the *Streptomyces* enzymes. Deletion of the disulfide bond in PETase significantly reduced enzyme activity [17]. Similarly, in the area of primary structure marked in light red, the PETase enzyme has additional residues compared to sequences A–E perhaps creating a larger active site which better accommodates PET [9]. Joo et al. [14] compared the PETase enzyme with that of *Thermobifida fusca* (both shown in Figure 2). Compared with the *T. fusca*-enzyme (and also with those from *Streptomyces*), PETase has an ‘extended loop’ [14], shaded grey in the figure, which is absent or shorter in the *Streptomyces* enzymes. The unique conformation of the extended loop in PETase has been shown to be necessary for the formation of some subsites of the substrate binding site. In addition, completely unique to PETase is the Ser(S) residue shown in bold and shaded dark red. The other sequences have the larger His (H) at this position. The relevance of this is as follows: the Trp(W) residue, also shaded red in the figure, is strictly conserved across all homologous sequences and may play a role in substrate binding [9]. However, it exhibits more than one conformation (wobble) exclusively in PETase. Trp(W) wobbling is closely related to the binding of substrate and product and it appears that His(H) and Trp(W) side chains would stack and restrict conformational change in the latter. Because it is smaller, the Ser(S) residue in PETase yields necessary space for Trp(W) rotation [9]. His(H) substitution for Ser(S) in PETase was shown experimentally to partially compromise PETase activity [17]. The above presentation shows that several critical features (e.g., additional disulfide bonds and the extended loop) contributing to the excellent PET-hydrolyzing activity of PETase are lacking in the *Streptomyces* sequences, and probably account for the lack of observed activity against amorphous PET in growth medium (see below). Figure 3 shows a similarity tree of the enzymes presented in Figure 2 and additional sequences.

The five *Streptomyces* enzymes (A–E) from the present study group together in their own clade. Sequences A/E and B/C were identical over the sequenced range, although all the isolates originate in different samples from different locations (Table 1 and Table 2). The phylogenetic tree shares some features with that produced by Joo et al. [14] for a larger number of possible PET-degrading enzymes, and suggests that the *Streptomyces* enzymes A–E belong in these authors’ class I group of PETase-like enzymes. Similarity searches on the A and E sequences using BLASTP (non-redundant database) produced essentially the same percentage identity score with *Streptomyces* dienelactone hydrolase (E.C.3.1.1.45) and ‘platelet-activating factor acetylhydrolase isoform II’ (a microbial lipase) families over the whole sequenced length. The top score for sequences B, C and D was an annotated ‘Multispecies: *Streptomyces* α/β hydrolases.’ Dienelactone hydrolase is a carboxylic α/β ester hydrolase which catalyzes the hydrolysis of dienelactone to maleyacetate and forms part of the beta-ketoadipate pathway used in bacteria and fungi to degrade aromatic compounds. However, using FASTA [22], to search only the manually annotated section of UniProtKB [23], the top hit for all sequences was a leaf-branch compost cutinase, LC-cutinase [55]. PETase from *I sakaienesis* was the second most likely identification. The gene encoding LC-cutinase comes from a compost metagenome; the source organism remains to be identified but is probably a thermophile. After cloning and expression, the enzyme product was shown to have PET-hydrolyzing ability [55]. Figure 3 shows that the LC-cutinase groups with a recently reported α/β-hydrolase identified in *Streptomyces* sp. SM14 isolated from a marine sponge [45]. Polyesterase activity was demonstrated using PCL as substrate, but possible activity on PET does not appear to have been investigated. 

Isolates A–E and the control *I. sakaiensis* were cultured in the presence of amorphous PET. After several weeks of incubation, the control strain grew round and discolored the plastic (results not shown). This result was not seen with the *Streptomyces* isolates, or a number of other bacteria, suggesting an effect on PET is absent or limited. It was also noteworthy that the presence of the gene in *Streptomyces* did not always correlate with the hydrolysis of PCL (Table 2). However, a more sensitive approach to assaying for low-level PET-hydrolysis activity, such as product formation, is justified based on similarity searches, and would be useful. In addition to the PETase-like enzyme identified in *Streptomyces* sp. SM14 discussed above [45], there is a recent report of a suberinase produced by *Streptomyces scabies* with the ability to degrade cutin and PET as well as suberin [56]. The genus *Streptomyces* should now be more deeply mined with high-resolution techniques with the aim of identifying PET-hydrolyzing and homologous enzymes.

The PCR/sequencing-based approached described in this paper is a rapid and useful technique for screening for potential PET-hydrolyzing enzymes. Extended studies are now desirable to understand the function of the gene-detected and its pattern of distribution in the genus. 

## 4. Conclusions

Although unlikely to be a final solution to problems associated with plastic waste, a phasing in of biobased biodegradable polymers may help to maintain the health of the environment. Various microbial strains have been detected that convert plastic polymers to monomers, which in turn may be subsequently completely metabolized. If it can be made to work in the environment or in industrial processes, the exploitation of microbes for degradation of plastics would be an eco-friendly method. A logical first step, represented by the present and other studies, is to identify the types and specificities of natural plastic-degrading microbes. Microbes and the enzymes that they produce represent a major area of current research in connection with a potential use in the bioremediation of plastic refuse. However, the diversity of known enzymes and microbes acting on synthetic polymers is still rather limited [7]. The current work extends this list with 11 new isolates. Furthermore, no studies on plastic-degrading bacteria isolated in Norway have to my knowledge been published previously. Norway has a generally cold climate, and although not investigated directly in the present work, its microbes might be better suited to bioremediation at lower temperatures. Sampling took place during the winter months in Norway. The present study shows that some isolates degrade not only multiple plastics, but also a range of other polymers (e.g., starch, cellulose) that have been used alone or in plastic blends. A *Rhodococcus* sp., but most especially a *Streptomyces* sp., may have a bioremediative potential. In addition to charting plastic-degrading capabilities, the present work also provides metabolic profiles for future reference. The study shows the presence of a gene coding a PETase-like enzyme in some *Streptomyces* isolates, which, given some very recent publications on plastic (including possibly PET)-degrading members of the genus, is worthy of further investigation.

## Figures and Tables

**Table 1:** Details on the nine sampling sites forming the core of the study.

Sample	Sampling Site	Coordinates	Sample Appearance	Additional Information
1	Soil from immediate vicinity of a small factory making plastic products to order.	59°40′12.8″N 10°47′45.0″E	Dry soil, with a dark appearance.	Sample was taken a few cm beneath the top soil.
2	Bank of a stream passing through an industrial area, close (20 m) to a small factory making plastic products to order	59°42′33.0″N 10°51′10.7″E	Brown, wet material apparent mix of soil and sand.	Sample was taken a few cm beneath the top layer
3	Natural marsh, several km from housing/industry.	59°34′12.1″N 10°40′17.7″E	Dark brown, watery sediment.	Sample taken under shallow water.
4	Bank of a stream running through housing estate and construction site.	59°33′51.1″N 10°44′02.6″E	Clay-like material.	Sample was taken a few cm beneath the top layer
5	Bank of a river passing through agricultural area.	59°36′34.1″N 10°45′51.2″E	Brown, watery material with plant debris.	Sample was taken a few cm beneath the top layer
6	Edge of a small ditch below the outlet of a drainage pipe probably leading water from housing area and surroundings.	59°36′30.6″N 10°45′55.4″E	Mixture of sand and clay-like material.	Sample was taken a few cm beneath the top layer
7	Bank of a stream about 100 m in the terrain below a sewage-treatment plant.	59°34′47.2″N 10°39′13.7″E	Light, finely-grained sand mixed with clay-like material.	Sample was taken a few cm beneath the top layer.
8	River bank in an urban area close to outlet to the Oslo fjord.	59°54′45.7″N 10°45′26.8″E	Coarse mix of sand and clay-like material.	Sample was taken a few cm beneath the top layer.
9	River bank in an urban/industrialized area.	59°54′46.8″N 10°49′40.7″E	Mix of sand and clay-like material.	Sample taken from top layer of sediment

**Table 2:** Polymer degrading profiles for environmental isolates.

Agar	Isolate i–xi (sample of origin 1–9;—Table 1)										
	i *	ii *	iii (1)	iv (9)	v (7)	vi (7)	vii (7)	viii (4)	ix (5)	x (5)	xi (3)
PHB	+	+	−	+	+	+	−	+	−	−	+
PHBV	+	+	+	+	+	+	−	+	+	−	+
PES	−	−	−	−	−	−	+	+	−	−	−
PBS	−	−	−	−	−	−	+	[+]	−	−	−
PCL	−	[+]	−	−	−	−	[+]	+	−	[+]	−
PLLA	−	−	−	−	−	−	−	−	−	−	−
RES	−	−	−	−	−	−	+	+	−	+	−
Starch (amylase)	−	+	−	+	+	−	−	+	+	−	+
CMC (cellulase)	−	+	+	+	+	−	−	+	‘	−	+
DNA (DNase)	−	−	−	−	−	−	−	−	−	−	−
Protein (protease)	−	+	−	−	−	−	−	+	−	+	−
Tributyrin (esterase)	+	+	+	[+]	+	−	+	+	+	−	+
Olive oil (lipase)	−	+	−	−	−	−	−	−	−	−	−
Coconut oil (lipase)	−	[+]	−	−	−	−	+	−	−	−	−
**Number +**	**3**	**9**	**3**	**5**	**5**	**2**	**6**	**10**	**3**	**3**	**5**
PCR product (PET-primers)	−	−	−	+	−	−	−	+	−	−	+

Roman numerals (i–xi) denote bacterial isolates. * Isolates i–ii were obtained from a previous investigation (see methods for their origin). Isolates iii–xi forming the core of the present study, were isolated from the samples shown in Table 1 as described in the methods section. [+] = weakly positive reaction.

**Table 3:** Identification of isolates based on partial sequencing of the 16S rDNA gene.

Isolate	Sequence Length (bp)	Phylogenetic Affiliation *	Closest Species (Accession Number)	Identity-% (Query Coverage)
i	625	*Acidovorax*	*A. facilis* LMG2193 (EU024133)	99.36 (100)
ii	526	*Streptomyces*	*S. similanensis* KC-106 ** (AB773850)	99.62 (100)
iii	508	*Streptomycetaceae*	*S. urticae* NEAU-PCY-1 (KY788226)	99.80 (100)
iv	540	*Streptomyces*	*S. lunaelactis* MM109 ** (KM207217)	100 (100)
v	592	*Streptomyces*	*S. alboniger* DSM40043 ** (AY845349)	99.49 (100)
vi	510	*Pseudomonas*	*P. floridensis* GEV388 ** (KY614191)	100 (100)
vii	731	*Rhodococcus*	*R. fascians* CF17 (X79186)	99.86 (100)
viii	1403	*Streptomyces*	*S. brevispora* BK160 (FR692104)	99.86 (100)
ix	528	*Oxalobacteraceae*	*Rugamonas rubra* MOM 28/2/79 (HM038005)	99.43 (99)
x	422	*Nakamurella*	*N. lactea* ** DSM19367 (HE599561)	98.82 (100)
xi	951	*Streptomyces*	*S. fulvissimus* DSM40593 **	100 (100)

* From RDP classifier. Lowest affiliation with a score of 1.0 at default 80% confidence setting. ** Where there are other strains or species with same total score.
